# *In vitro* selection of cefiderocol-resistant mutants in *Acinetobacter baumannii* harbouring the most common carbapenemase genes

**DOI:** 10.1093/jac/dkaf462

**Published:** 2025-12-17

**Authors:** Otávio Hallal Ferreira Raro, Jacqueline Findlay, Laurent Poirel, Jean-Winoc Decousser, Patrice Nordmann

**Affiliations:** Medical and Molecular Microbiology, Faculty of Science and Medicine, University of Fribourg, Chemin du Musée 18, Fribourg CH-1700, Switzerland; Medical and Molecular Microbiology, Faculty of Science and Medicine, University of Fribourg, Chemin du Musée 18, Fribourg CH-1700, Switzerland; Medical and Molecular Microbiology, Faculty of Science and Medicine, University of Fribourg, Chemin du Musée 18, Fribourg CH-1700, Switzerland; Swiss National Reference Center for Emerging Antibiotic Resistance, University of Fribourg, Fribourg, Switzerland; Microbiology Unit, University Hospital Henri Mondor, Assistance Publique-Hôpitaux de Paris, Créteil, France; Health Faculty, DYNAMYC EA 7380, University Paris Est Créteil, Créteil, France; Medical and Molecular Microbiology, Faculty of Science and Medicine, University of Fribourg, Chemin du Musée 18, Fribourg CH-1700, Switzerland; Swiss National Reference Center for Emerging Antibiotic Resistance, University of Fribourg, Fribourg, Switzerland

## Abstract

**Background:**

Carbapenem-resistant *Acinetobacter baumannii* (CRAB) represents a critical global health threat due to its high mortality rates in infections, limited treatment options and resistance to last-resort antibiotics. Cefiderocol, a novel siderophore-conjugated cephalosporin, has emerged as a promising therapeutic agent against such infections. However, resistance to this antibiotic has already been reported.

**Objectives:**

To investigate whether the production of common carbapenemases affects cefiderocol susceptibility in *A. baumannii* and to identify the genetic mechanisms underlying resistance development.

**Methods:**

Using isogenic *A. baumannii* CIP 7010 strains carrying carbapenemase genes commonly identified in that species, namely *bla*_NDM-1_, *bla*_OXA-23_, *bla*_OXA-40_ or *bla*_OXA-58_, mutants were selected under increasing cefiderocol pressure. Mutants obtained were subjected to MIC determinations, WGS, complementation assays, real-time quantitative PCR (RT-qPCR) and fitness assays.

**Results:**

WGS of cefiderocol-resistant isolates revealed recurrent mutations in genes encoding the global regulators BfmS and OxyR, rather than in genes directly related to iron uptake, PBPs or β-lactamases. Complementation assays with WT *bfmRS* or *oxyR* resulted in a reversion to the parental strain cefiderocol MICs. RT-qPCR indicated that these global regulators reduced expression of *piuA* and *pirA* genes involved in iron uptake, and hence were associated with a previously unknown mechanism that results in resistance to cefiderocol.

**Conclusions:**

Global regulators BfmS and OxyR were responsible for decreasing the expression of *piuA* and *pirA* genes, thereby contributing to cefiderocol resistance. This evolutionary analysis enhances our understanding of the mechanisms underlying cefiderocol resistance and identifies potential molecular targets for the development of new therapeutics against CRAB.

## Introduction

The ability of *Acinetobacter baumannii* to cause severe human infections, acquire antimicrobial resistance genes and persist in hospital environments, has led to carbapenem-resistant *A. baumannii* (CRAB) being classified as ‘critical’ in the WHO bacterial priority pathogens list for research and development of new medicines, diagnostics and prevention tools.^1^

CRAB strains are responsible for a wide range of infections including hospital- and community-acquired pneumonia, bloodstream infections, urinary tract infections and wound infections. These infections often progress to severe stages among intensive care patients, particularly in low- to middle-income countries, leading to alarmingly high mortality rates.^1–3^ CRAB was listed among the six leading pathogens associated with deaths due to antimicrobial resistance in 2019 and 2021, with attributable mortality of 57 700 and 78 100 deaths, respectively.^2,4^

Despite the critical threat posed by CRAB, the high morbidity and mortality, and the increased reports of resistance to clinically relevant antibiotics, therapeutic options remain severely limited, and antibiotic development has stagnated. Notably, no clinically effective agents are currently available against MBL-producing CRAB strains, underscoring the urgent need for sustained investment in antimicrobial research and development.^5,6^

The management of CRAB infections remains challenging due to the toxicity and limited efficacy of last-resort agents. Agents such as colistin and polymyxin B are frequently associated with nephrotoxicity, and tigecycline is associated with poor penetration in infection sites like the lungs and the urinary tract.^7^ The recently developed sulbactam/durlobactam combination shows promise; however it is ineffective against MBL-producing strains, leaving a critical therapeutic gap.^8^

These challenges highlight the urgent need for novel antimicrobial agents active against resistant *A. baumannii*. Cefiderocol, a siderophore-conjugated cephalosporin, represents a novel therapeutic approach that exploits the bacterial iron uptake systems. It binds extracellular iron and is actively transported into Gram-negative bacteria via catecholate siderophore receptors, while also diffusing through outer membrane porins thus enhancing access to target sites into the periplasmic space of the cell.^9^ Cefiderocol has been approved for treating adults with complicated Gram-negative urinary tract infections, hospital-acquired pneumonia and ventilator-associated pneumonia.^9,10^

However, infections caused by cefiderocol-resistant CRAB strains have already been reported. Acquired resistance has been associated with mutations in genes involved in iron acquisition, including *pirA* (siderophore gene) and *piuA* (TonB-dependent siderophore receptor), β-lactamases genes (NDM-like, SHV-12 and PER-like), and point mutations in PBP1a and PBP3.^11–14^ To better understand the influence of carbapenemase types on cefiderocol resistance in *A*. *baumannii*, our study aimed to select cefiderocol-resistant mutants from a collection of isogenic recombinant *A. baumannii* CIP 7010 isolates carrying the most frequently acquired carbapenemase genes in this species, namely *bla*_NDM-1_, *bla*_OXA-23_, *bla*_OXA-40_ or *bla*_OXA-58_, and to identify the mechanisms responsible for resistance development.

## Materials and methods

### Construction of the A. baumannii CIP 7010 library containing carbapenemase genes

The strain *A. baumannii* CIP 7010 (ATCC 15151) was selected for this study because it is a fully sequenced, antibiotic-susceptible reference strain of *A. baumannii* commonly used in comparative genomics and resistance mechanism studies.^15^ To generate the isogenic recombinant strains, primers were used to amplify *bla*_NDM-1_, *bla*_OXA-23_, *bla*_OXA-40_ or *bla*_OXA-58_, the resulting products were ligated into the shuttle vector pVRL1,^16^ and transformed into *Escherichia coli* TOP10, using 15 mg/L gentamicin for selection. Plasmids were then extracted using Zyppy Plasmid Miniprep Kit (Zymo Research, Irvine, CA, USA) and transformed into *A. baumanniii* CIP 7010 by electroporation. Primers are shown in Table S1 (available as Supplementary data at *JAC* Online).

### Susceptibility testing

Susceptibility testing was performed by broth microdilution using an in-house-prepared iron-depleted Mueller–Hinton broth (ID-MHB) to determine the MIC of cefiderocol. MHB was used for all the other antibiotics for all isolates according to the EUCAST guidelines.^17,18^ Interpretation of the results for cefiderocol was performed in accordance with the pharmacokinetic/pharmacodynamic breakpoints from EUCAST (susceptible ≤2 mg/L; resistant >2 mg/L).^17,18^ Since there is a lack of breakpoints available for *A. baumannii*, the reference strains *E. coli* ATCC 25922, *Pseudomonas aeruginosa* ATCC 27853 and *A*. *baumannii* NCTC 13304^19^ were used for broth microdilution (BMD) quality control.

### Selection of cefiderocol-resistant mutants

Cefiderocol-resistant *A. baumannii* mutants were selected in ID-MHB using several passages with increasing concentration of cefiderocol. Briefly, a single colony of each strain was first grown in LB broth overnight at 37°C without antibiotics. The strains were then inoculated in a 1:100 ratio in 5 mL of ID-MHB, with an initial concentration of cefiderocol of 0.5 mg/L, for 24 h. Then strains were plated onto LB agar plates, and two single colonies were recovered and stored at −80°C. Additionally, from the remaining broth with 0.5 mg/mL of cefiderocol, the strains were transferred (1:100) to 5 mL of fresh media containing double the initial concentration of cefiderocol. After that, both processes (single colony recovery and increasing cefiderocol concentration) were repeated every 24 h until no further bacterial growth was observed in both LB broth and agar.

### WGS

Genomic DNA (gDNA) was isolated from overnight-grown bacterial cultures using the QIAamp DNA Mini Kit (Qiagen, Valencia, CA, USA). WGS was carried out using the Illumina MiSeq (San Diego, CA, USA) platform. Libraries were prepared with the Nextera DNA sample preparation protocol, generating 2 × 300 bp paired-end reads with a minimum depth of 30× coverage. Short-read data were quality-trimmed using Trimmomatic and assembled with the Shovill pipeline (https://github.com/tseemann/shovill).^20^ Resulting contigs were annotated using Prokka.^21^ Resistance gene detection and species confirmation were conducted via the Center for Genomic Epidemiology’s tools: ResFinder 4.1 and KmerFinder 3.2 (https://www.genomicepidemiology.org/).^22,23^ Snippy (https://github.com/tseemann/snippy) was used to search for substitutions such as SNPs and multiple nucleotide polymorphisms, and for insertions or deletions between the reference genome (*A. baumannii* CIP 7010 plus the pVRL1 empty vector) and the mutant isolates. Sequencing datasets from this study were deposited in the NCBI Sequence Read Archive under BioProject accession number PRJNA1338351.

### bfmRS and oxyR cloning and complementation

Wild-type *bfmRS* and *oxyR* were amplified from *A. baumannii* CIP 7010 and cloned into pUBYT using the primers listed in Table S1,^24^ to generate plasmids pUBYT-*bfmRS* and pUBYT-*oxyR*. Both the original plasmid, pUBYT, and recombinant plasmids were electroporated into strains and mutants, with selection on LB agar supplemented with 50 mg/L kanamycin.

### mRNA extraction and cDNA synthesis

Strains were inoculated in ID-MHB and incubated at 37°C with shaking until reaching the OD of 0.5. The total RNA was extracted using the Quick-RNA MiniPrep kit (Zymo Research, Irvine, CA, USA). To remove contaminating DNA from RNA preparations, the Turbo DNA-free kit (Invitrogen, Waltham, MA, USA) was used. cDNA synthesis was performed by using LunaScript RT SuperMix kit (New England BioLabs, Ipswich, MA, USA). cDNA sample concentrations were measured using the NanoDrop 2000 spectrophotometer (Thermo Fisher Scientific, Waltham, MA, USA).

### RT-qPCR

Quantitative real-time PCR experiments were performed using the Rotor-Gene Q cycler (Qiagen, Hilden, Germany). The primers used in the experiments were targeting the genes *rpoB* (reference), *piuA* and *pirA* (primers are shown in Table S1). Reactions were set up in a total volume of 20 µL with a GoTaq qPCR Master Mix kit (Promega, Madison, WI, USA). The cycle threshold values were analysed by the 2^−ΔΔCT^ method.^25^ Relative expression levels were calculated by comparison with the control samples, and the condition values were corrected with the appropriate reference gene. Experiments were performed on three independent biological replicates with two technical replicates of each reaction. Data were analysed by one-way analysis of variance (ANOVA) corrected for multiple comparisons using statistical hypothesis testing with Tukey using GraphPad Prism Software version 9.5.1. Statistically significant results were defined as *P* value <0.05.

### Fitness evaluation

Measurements of growth rates were performed with the *A*. *baumannii* CIP 7010 strain in comparison with the mutated versions of the respective genes obtained after *in vivo* selection with cefiderocol. Growth curves were obtained following standard protocols and performed in ID-MHB in 96-well microplates. Bacterial growth was accessed by measuring OD at 600 nm wavelength (OD_600_) using a BioTek Cytation 5 plate reader (Agilent Technologies, Santa Clara, CA, USA) over a 24 h period. Experiments were performed on three independent replicates with two technical replicates of each. Statistical analyses were conducted using two-tailed parametric *t*-tests, and statistical significance was defined as a *P* value <0.05. Analyses were performed using GraphPad Prism version 9.5.1.

## Results and discussion

For cefiderocol mutants obtained from each genetic construct, three independent colonies were initially selected for sequencing and antimicrobial susceptibility testing. Colonies that displayed identical resistance profiles and mutations were considered equivalent and represented by only a single isolate or at most two per strain to avoid redundancy and facilitate data interpretation. According to this selection process and the variable selective endpoints, the following mutants were chosen: one mutant carrying the empty vector pVRL1 (pVRL1), one with pVRL1 harbouring *bla*_NDM-1_ (pVRL1 + NDM-1), one with pVRL1 harbouring *bla*_OXA-23_ (pVRL1 + OXA-23), and one with pVRL1 harbouring *bla*_OXA-58_ (pVRL1 + OXA-58a), all recovered at 8 mg/L of cefiderocol. Additionally, mutants carrying pVRL1 with *bla*_OXA-40_ (pVRL1 + OXA-40) and a second colony with *bla*_OXA-58_ (pVRL1 + OXA-58b), were selected at 64 mg/L of cefiderocol. The MIC values and corresponding mutations for all selected mutants are summarized in Table 1. The obtained mutations conferred resistance to cefiderocol without cross-resistance to the other antibiotics tested. This was particularly evident for antibiotics currently considered potential alternative treatments for infections caused by MDR *A. baumannii*, namely ampicillin/sulbactam, sulbactam/durlobactam and colistin according to the IDSA.^26^

**Table 1.** MICs of several antibiotics for parental and mutant strains and protein sequence changes in BfmS and OxyR

Strain	FDC selection, mg/L	Protein sequence changes		MIC, mg/L											
		BfmS	OxyR	FDC	PIP	CAZ	FEP	IPM	MEM	SAM^a^	SUL/DUR^a^	CST	TET	CIP	AMK
CIP 7010	—	—	—	0.12	16	1	1	≤	0.25	2	≤0.12	≤0.12	0.5	≤0.12	1
CIP 7010 + pVRL1	—	—	—	0.12	16	1	1	0.12	0.25	2	0.5	≤0.12	0.25	≤0.12	1
CIP 7010 + pVRL1	8	Ala321Glu	—	>128	16	1	1	0.12	0.25	2	0.5	≤0.12	0.5	0.25	1
CIP 7010 + pVRL1 + NDM-1	—	—	—	1	128	>128	32	4	8	16	8	≤0.12	0.5	≤0.12	1
CIP 7010 + pVRL1 + NDM-1	8	Ala321Glu	—	>128	>128	>128	32	8	8	16	8	≤0.12	0.5	≤0.12	1
CIP 7010 + pVRL1 + OXA-23	—	—	—	0.12	>128	2	2	2	2	8	1	≤0.12	0.25	≤0.12	1
CIP 7010 + pVRL1 + OXA-23	8	—	Leu197Arg	>128	>128	2	2	2	2	8	1	≤0.12	0.25	≤0.12	1
CIP 7010 + pVRL1 + OXA-40	—	—	—	0.12	>128	2	2	0.5	1	8	1	≤0.12	0.25	≤0.12	1
CIP 7010 + pVRL1 + OXA-40	64	—	Val52f/s^b^	>128	>128	2	2	0.5	1	8	1	≤0.12	0.25	≤0.12	1
CIP 7010 + pVRL1 + OXA-58	—	—	—	0.12	>128	2	2	0.5	1	8	1	≤0.12	0.25	≤0.12	1
CIP 7010 + pVRL1 + OXA-58	8	Ile80^b^	—	>128	>128	2	2	0.5	1	8	1	≤0.12	0.25	≤0.12	1
CIP 7010 + pVRL1 + OXA-58	64	—	Ser174f/s^b^	>128	>128	2	2	0.5	1	8	1	≤0.12	0.25	≤0.12	1

AMK, amikacin; AZT, aztreonam; CAZ, ceftazidime; CIP, ciprofloxacin; CST, colistin; FDC, cefiderocol; FEP, cefepime; f/s, frameshift; IPM, imipenem; MEM, meropenem; PIP, piperacillin; SAM, ampicillin/sulbactam; SUL/DUR, sulbactam/durlobactam; TET, tetracycline.

^a^Fixed concentration of 4 mg/L.

^b^Stop codon.

Genes encoding the global regulators BfmS or OxyR were regularly altered across several cefiderocol-resistant mutants, regardless of the carbapenamase carried. Two distinct alterations were observed in BfmS across different parental stains: Ala231Glu (pVRL1-derived and NDM-1-derived) and Ile80-stop [stop codon; (OXA-58-derived)]. Similarly, three other differences were also observed in OxyR: Leu197Arg (OXA-23-derived), Val52f/s-stop [f/s, frameshift (OXA-40-derived)] and Ser174f/s-stop (OXA-58-derived). All six mutants harbouring mutations in either *oxyR* or *bfmS* exhibited cefiderocol MIC >128 mg/L (Table 1). The same types of mutations in the *bfmS* and *oxyR* regulatory genes were obtained regardless of the carbapenemase gene content. Therefore, the expression of the NDM gene did not influence the type of mutations obtained, although it has been reported as a favourable background for the development of reduced susceptibility to cefiderocol.^11^

Complementation assays were performed by introducing WT *bfmRS* or *oxyR* into one representative of each mutation, resulting in five cefiderocol-resistant mutants being tested to assess any impact on MICs of cefiderocol. In all five mutants a significant decrease in cefiderocol MICs to parental MICs was observed in most cases (Table 2). These results confirmed that the identified mutations directly impacted cefiderocol susceptibility. Notably, the presence of the different carbapenemase enzymes did not influence the level of cefiderocol resistance among the mutants.

**Table 2.** MICs of cefiderocol for mutants obtained at several cefiderocol concentrations and complemented with WT *bfmRS* or *oxyR* genes

Strain	Protein sequence changes	FDC MIC, mg/L
pVRL1–8 mg/L	BfmS—Ala321Glu	>128
pVRL1–8 mg/L/pUBYT		>128
pVRL1–8 mg/L/pUBYT-*bfmRS*		1
OXA-58–8 mg/L	BfmS—Ile80^a^	>128
OXA-58–8 mg/LpUBYT		≥128
OXA-58–8 mg/LpUBYT-*bfmRS*		2
OXA-23–8 mgL	OxyR—Leu197Arg	>128
OXA-23–8 mg/L/pUBYT		≥128
OXA-23–8 mg/LpUBYT-*oxyR*		0.5
OXA-40–64 mg/L	OxyR—Val52f/s^a^	>128
OXA-40–64 mg/LpUBYT		>128
OXA-40–64 mg/LpUBYT-*oxyR*		0.5
OXA-58–64 mg/L	OxyR—Ser174f/s^a^	>128
OXA-58–64 mg/LpUBYT		≥128
OXA-58–64 mg/LpUBYT-*oxyR*		0.5

FDC, cefiderocol; f/s, frameshift.

^a^Stop codon.

RT-qPCR assays were then performed to evaluate the impact of these mutants on the expression of genes involved in iron acquisition in *A. baumannii*. The WT *A*. *baumannii* CIP 7010 strain served as a control, whereas one representative of each *oxyR* or *bfmS* mutation was analysed for the expression of the main iron transport genes, namely *piuA* and *pirA*. A statistically significant reduction in *piuA* and *pirA* expression was observed for all mutants compared with the control strain. Using *A*. *baumannii* CIP 7010 as the control (2^−ΔΔCT^: 1), *piuA* expression ranged from 0.03 to 0.07 in *oxyR* mutants and from 0.37 to 0.51 in *bfmS* mutants whereas *pirA* expression ranged from 0.09 to 0.37 and 0.12 to 0.42, respectively (Table 3).

**Table 3.** Relative expression of genes associated with iron transport and binding in cefiderocol-resistant *A. baumannii* strains in comparison with the control strain CIP 7010

Strain	FDC selection, mg/L	Protein sequence changes	2^−ΔΔCT^	
			*piuA*	*pirA*
CIP 7010	—	—	1	1
pVRL1	8	BfmS—Ala321Glu	**0.51** ± **0.21**	**0.42** ± **0.29**
OXA-58	8	BfmS—Ile80^a^	**0.37** ± **0.12**	**0.12** ± **0.06**
OXA-23	8	OxyR—Leu197Arg	**0.03** ± **0.01**	**0.37** ± **0.13**
OXA-40	64	OxyR—Val52f/s^a^	**0.07** ± **0.08**	**0.09** ± **0.06**
OXA-58	64	OxyR—Ser174f/s^a^	**0.03** ± **0.01**	**0.26** ± **0.11**

2^−ΔΔCT^, delta-delta CT method.

Bold indicates statistically significant results (*P* value < 0.05).

FDC, cefiderocol; f/s, frameshift.

^a^Stop codon.

These findings confirm that mutations in *oxyR* and *bfmS* indirectly promote cefiderocol resistance in *A. baumannii* by down-regulating the expression of a siderophore gene (*pirA*) and a TonB-dependent siderophore receptor (*piuA*), both previously associated with cefiderocol resistance.^13,14^ In contrast to prior reports describing direct substitutions in iron transporters *piuA* (Val216Gly, Thr280Ser, Asn381Ser, Ser412Thr) and *pirA* (Leu275Phe, I277Val),^27^ our study identified mutations in the regulatory genes *oxyR* and *bfmS* that indirectly reduce *pirA* and *piuA* expression. Similar mutations in iron transporters have also been linked to cefiderocol resistance in other species such as *P. aeruginosa* (e.g. *piuA*, *piuC*, *pirA*, *pvdS*) and Enterobacterales (e.g. *cirA*, *fiuA*).^28–30^

BfmS is a sensor kinase component of the two-component regulatory system BfmRS, which modulates virulence, antimicrobial resistance and environmental adaptation in *A. baumannii*.^31^ Geisinger *et al*.^32^ showed that *A. baumannii* lacking *bfmS* (Δ*bfmS*) exhibit increased resistance to several β-lactams, including aztreonam, ampicillin, carbenicillin, cephalexin, ceftazidime, mecillinam and sulbactam. However, in our study, *bfmS* mutants did not display increased MICs to β-lactams, suggesting no global cross-resistance effect. This supports the hypothesis that the observed increase in cefiderocol MICs is specifically related to impaired drug entry mediated by iron transport pathways involving *piuA* and *pirA*. Additionally, another two-component regulatory system, BaeSR, has been previously reported to mediate cefiderocol resistance.^33^ Liu *et al*.^33^ showed that BaeSR mutations promote resistance through strong up-regulation of the MacAB-TolC and an MFS efflux pump and activation of the phenylacetic acid operon (*paa)*. Both BfmRS and BaeSR systems modulate envelope-stress responses, yet they employ distinct downstream effectors: whereas the first limits drug entry, the second promotes drug efflux, highlighting a parallel regulatory resistance mediation with mechanistic divergence.^32,33^

OxyR is a LysR-type transcriptional regulator that functions as a master activator of the oxidative stress response, detecting and detoxifying hydrogen peroxide (H_2_O_2_) via alkyl hydroperoxide reductase (Ahp) and catalase (Kat), converting H_2_O_2_ to water.^34^ In *A. baumannii*, OxyR contributes to survival, virulence and genetic mobility (integrons or IS elements) through SOS and DNA repair responses.^35,36^ Inactivation of OxyR can trigger compensatory activation of other regulators (SoxR, RpoS, MarA), restoring oxidative stress tolerance and enhancing antibiotic resistance through efflux, envelope remodelling or DNA repair mechanisms.^37,38^ Although no previous studies have shown a direct role of OxyR in regulating iron uptake in *A. baumannii*, OxyR in *E. coli* has been reported to induce transcription of *dps*, which encodes a ferritin-like protein that protects DNA by sequestering iron.^39^ Moreover, an OxyR ortholog in *Cupriavidus pinatubonensis* positively regulates the type VI secretion system (T6SS1) by binding its promoter region, promoting iron import under oxidative stress.^40^

**Figure 1.**
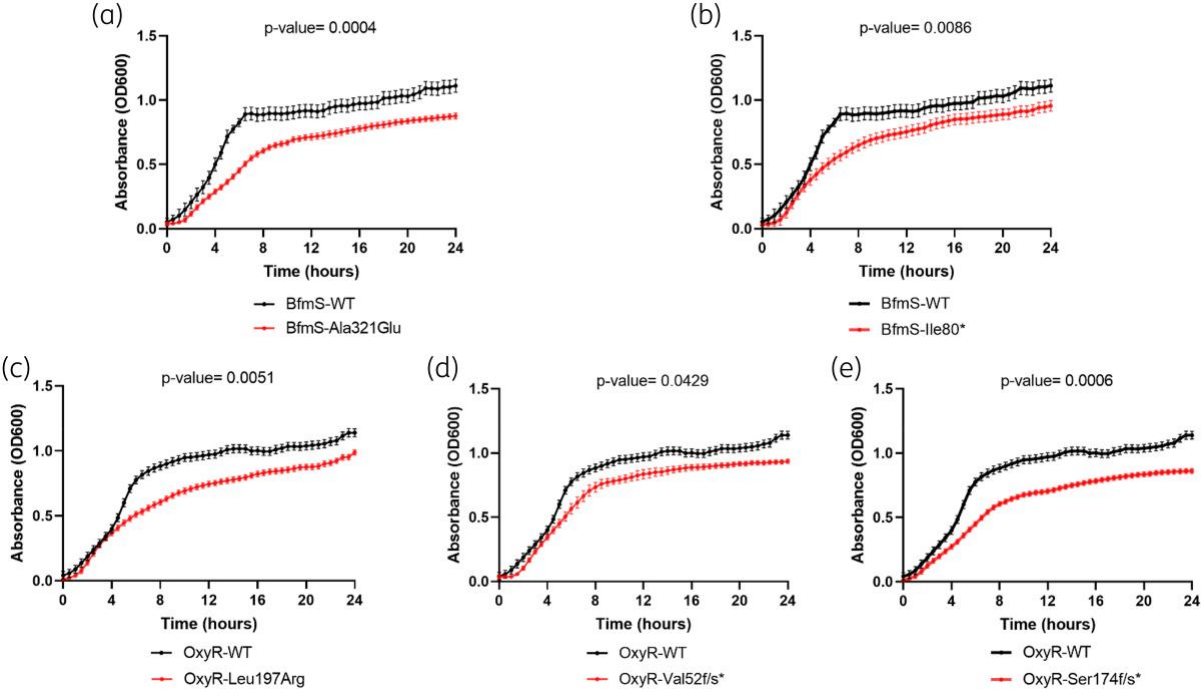
Growth curves of the BfmS-WT and OxyR-WT *Acinetobacter baumannii* CIP7010 and mutant isolates. (a) BfmS-WT versus BfmS-Ala321Glu. (b) BfmS-WT versus BfmS-Ile80*. (c) OxyR-WT versus OxyR-Leu197Arg. (d) OxyR-WT versus OxyR-Val52f/s*. (e) OxyR-WT versus OxyR-Ser174f/s*. Data are shown as mean ± SD. f/s, frameshift; *, stop codon.

Growth curve analyses revealed that mutations in either *bfmS* or *oxyR* significantly reduced bacterial fitness compared with WT strains (Figure 1). Although no direct evidence links BfmS or OxyR activation to iron availability in *A. baumannii*, our observations suggest that mutants harbouring these mutations may have an increased dependence on iron to maintain regulatory and stress-response pathways. Under limited iron availability in ID-MHB medium, these mutants are likely to face higher energetic demands to sustain regulatory activity, explaining their impaired growth.

BfmR, the response regulator of the BfmRS system, is negatively regulated by BfmS and directly controls genes involved in siderophore biosynthesis and transport.^41^ Given the essential role of iron in cellular function, our findings align with the hypothesis that restricted iron acquisition diminishes cell fitness.^42^ Additionally, OxyR has been shown to influence bacterial fitness and virulence: *A. baumannii* Δ*oxyR* strains are less abundant and less capable of disseminating in the liver and lungs of infected mice compared with WT *oxyR* strains.^43^

### Conclusions

Despite being crucial for metabolic and regulatory processes in *A. baumannii*, BfmS and OxyR have not been previously associated with resistance to cefiderocol. In this study, we demonstrated that the global regulators BfmS and OxyR are responsible for decreasing the expression of *piuA* and *pirA* genes, thereby contributing to cefiderocol resistance. This evolutionary analysis enhances our understanding of the mechanisms underlying cefiderocol resistance and identifies potential molecular targets for the development of new therapeutics against CRAB.

## Supplementary Material

dkaf462_Supplementary_Data

## Supplementary data

Table S1 is available as Supplementary data at *JAC* Online.
