# Supplementary material for: In vitro selection of cefiderocol-resistant mutants in Acinetobacter baumannii harbouring the most common carbapenemase genes
Source: J Antimicrob Chemother. 2025 Dec 17;81(1):dkaf462. doi: 10.1093/jac/dkaf462 (PMC12802952; doi:10.1093/jac/dkaf462)
Supplement: dkaf462_Supplementary_Data [file dkaf462_supplementary_data.docx]

| **Primer** | **Sequence (5’-3’)** |
| --- | --- |
| NDM_BamHIF | GATGATGGATCCTCATCTATTTACTAGGCCTCGCA |
| NDM_SacIR | GATGATGAGCTCCGCATAAAACGCCTCTGTCA |
| OXA_23_BamHIF | GATGATGGATCCGTCGTGTACAGAGTTATTTTCT |
| OXA_23_SacIR | GATGATGAGCTCAGTCAGATTATAAAAGGCCCA |
| OXA_24_40_BamHIF | GATGATGGATCCGCCCCAAAATTTCCCCTAACA |
| OXA_24_40_SacIR | GATGATGAGCTCTTCGGGCCAAACTGAGAAAC |
| OXA_58_BamHIF | GATGATGGATCCTTGCTAGAGTTATTTGCATT |
| OXA_58_SacIR | GATGATGAGCTCAGATTAACCTCAAACTTCTA |
| bfmRS_BamHIF | GATGATGGATCCGCATAGCTCCATTGTCACTTTTG |
| bfmRS_NotIR | GATGATGCGGCCGCCTGAGAAGCGGCAAACCTAT |
| oxyR_BamHIF | GATGATGGATCCCTTACTGCAACTGGACCGTG |
| oxyR_NotIR | GATGATGCGGCCGCACTTGAGATCGGCTTGGGTA |
| qPCR_rpoB_qF | ACGCCTAAAGGTGAAACTCAGTTAA |
| qPCR_rpoB_qR | GTACCAGATGGAACACGTAAAGATG |
| qPCR_piuA_qF | CAGTTGGTGGCAG CATCAAT |
| qPCR_piuA_qR | TGCTGCAATGCCATTTCCAA |
| qPCR_pirA_qF | GGTTCAGGAGGTGCGAATTC |
| qPCR_pirA_qR | ATTACTCACACCAACACGCG |
| qPCR_fur1_qF | ACAACATCATCTTAGCGCCG |
| qPCR_fur1_qR | CCCGCAGCTTCAAATTGTGT |

**Table S1.** Primers used for clonings into vectors pVRL1 and pUBYT, and for RT-qPCR experiments.
